# The Yin–Yang of Microvesicles (Exosomes) in Cancer Biology

**DOI:** 10.3389/fonc.2014.00172

**Published:** 2014-07-09

**Authors:** Khalid Al-Nedawi

**Affiliations:** ^1^Department of Medicine, McMaster University, Hamilton, ON, Canada; ^2^St. Joseph’s Healthcare, Hamilton, ON, Canada

**Keywords:** microvessels, exosomes, PTEN, metastasis, progression

Microvesicles and exosomes have emerged as a new mode of intercellular communication in cancer. In recent years, microvesicles have received increasing attention from the scientific community for their role in regulating and transferring active molecules responsible for tumor progression and metastasis. Controversy arises from the fact that microvesicles can transfer tumor-promoting molecules such as oncoproteins (1–3), and tumor suppressors (4, 5). So, how can we make sense of this controversy?

By considering what we know of cancer development, we can rectify the seemingly opposing findings of the role of microvesicles in this process. The role of oncogenes in the progression of cancer is a well-investigated matter, and it is known that cancer progression is dependent on overexpression or mutation of oncogenic proteins. On the other hand, it also depends on loss or downregulation of tumor suppressor proteins.

The effect of the intercellular exchange of oncoproteins and tumor suppressor proteins through microvesicles on cancer progression most probably follows the “Yin–Yang” concept. The availability of microvesicles rich in oncoproteins or those containing tumor suppressors will decide the new phenotype characteristics acquired by the acceptor cells. In the case of the tumor suppressor PTEN, it is interesting to note that metastases often have no expression of PTEN, although the primary tumor itself expresses PTEN (6). This may indicate that cells capable of initiating metastasis originate from cellular selection (7), whereby cells with little or no expression of PTEN are more likely to initiate successful metastases (8, 9). Furthermore, it is known from a series of studies of two tumor systems that tumors “talk” to each other through serum-borne factors (10–12). A tumor at one side of an experimental animal can affect the dynamics of a tumor on the other side. However, the serum-borne factors suggested in these studies are not fully defined; microvesicles represent a reasonable candidate as a player in this form of cellular communication. Such a mode of cellular communication may have a role in tumor metastasis.

Microvesicles that contain PTEN are shed from primary PTEN-expressing tumors to the bloodstream, and may represent the serum-borne factors that are used by the primary tumor to affect the growth of metastases not expressing PTEN. This may indicate a successfully adopted mechanism throughout the natural history of cancer development, in which PTEN-null cells in the primary tumor form “tumor’s progeny” in the form of metastases. These metastases may be more aggressive than the primary tumor, because they do not express PTEN. The primary tumor then controls the growth of its “progeny” through tumor suppressor (PTEN) enriched microvesicles, which circulate in the blood and keep PTEN-null metastases in dormancy by supplementing them with PTEN protein. This hypothesis may explain the known phenomenon in which removal of the primary tumor enhances the growth of metastases, because the source of the PTEN-bearing microvesicles is removed.

On the other hand, microvesicles enriched with oncogenic proteins may be shed by tumor cells to oppose the increased metabolic demand associated with overexpression of oncogenic proteins. Oncogenic protein expressing microvesicles are also used as intercellular mediators to engage other cells (which may be healthy) to provide a suitable growth niche to nourish cancer cells. For example, EGFR-bearing microvesicles stimulate endothelial cells to secrete vascular endothelial growth factor (VEGF) and the autophosphorylation of the VEGF receptor-2 (VEGFR2), triggering the angiogenic switch to initiate angiogenesis (1).

It is noteworthy that microvesicles also contribute to the transfer of other molecules such as mRNAs and miRNA (miR), which can have various effects on tumor progression by modulating tumor microenvironment. An interesting cross-talk was found between hepatocarcinoma cells, whereby cells expressing miR-122 send this miR via microvesicles to inhibit the proliferation of miR-122 deficient cells. In a reciprocal process, miR-122 deficient cells secrete insulin-like growth factor to decrease miR-122 expression in miR-122 expressing cells (13). Microvesicles from cancer cells are found to stimulate tumor-associated macrophages to secrete VEGF, through the transfer of miR-150 (14) and thereby enhance tumorigenicity. In an opposite effect, macrophages inhibit proliferation of hepatocarcinoma cells by transferring miR-142 and miR-223 via microvesicles (15). A similar effect is found for microvesicles from marrow stromal cells expressing miR-146, which inhibit the proliferation of glioma cells (16).

In this sense, the role of microvesicles that contain molecules whose effects are seemingly in opposition (oncoproteins, oncomirs, or tumor suppressors) may be reconciled from the perspective of the tumor, a successful parasitic organ generated from our own cells. We realize that this commentary may contain some speculations, but we hope that it can trigger serious discussions in tumor biology, which may contribute to the long battle against the killer known as cancer.

## Conflict of Interest Statement

The author declares that the research was conducted in the absence of any commercial or financial relationships that could be construed as a potential conflict of interest.
